# Collagen fibers provide guidance cues for capillary regrowth during regenerative angiogenesis in zebrafish

**DOI:** 10.1038/s41598-021-98852-6

**Published:** 2021-09-30

**Authors:** Anita Senk, Valentin Djonov

**Affiliations:** 1 Institute of Anatomy, University of Bern, Bern, Switzerland; 2 Graduate School for Cellular and Biomedical Sciences, University of Bern, Bern, Switzerland

**Keywords:** Angiogenesis, Cell migration

## Abstract

Although well investigated, the importance of collagen fibers in supporting angiogenesis is not well understood. In this study, we demonstrate that extracellular collagen fibers provide guidance cues for endothelial cell migration during regenerative angiogenesis in the caudal zebrafish fin. Inhibition of collagen cross-linking by β-Aminopropionitrile results in a 70% shorter regeneration area with 50% reduced vessel growth and disintegrated collagen fibers. The disrupted collagen scaffold impedes endothelial cell migration and induces formation of abnormal angioma-like blood vessels. Treatment of the Fli//colRN zebrafish line with the prodrug Nifurpirinol, which selectively damages the active collagen-producing 1α2 cells, reduced the regeneration area and vascular growth by 50% with wider, but less inter-connected, capillary segments. The regenerated area contained larger vessels partially covered by endothelial cells embedded in atypical extracellular matrix containing cell debris and apoptotic bodies, macrophages and granulocytes. Similar experiments performed in early embryonic zebrafish suggested that collagens are important also during embryonic angiogenesis. In vitro assays revealed that collagen I allows for the most efficient endothelial cell migration, followed by collagen IV relative to the complete absence of exogenous matrix support. Our data demonstrates severe vascular defects and restricted fin regeneration when collagens are impaired. Collagen I therefore, provides support and guidance for endothelial cell migration while collagen IV is responsible for proper lumen formation and vascular integrity.

## Introduction

Angiogenesis, the formation of new blood vessels from pre-existing ones, is a fundamental requirement for several physiological and pathological processes. It occurs during tissue repair, expansion and remodeling throughout the physiological processes of development and maturation, wound healing and reproduction. It is also observed in pathological states of inflammation, autoimmune diseases, atherosclerosis and cancer^1–6^, all of which are principle contributors to mortality, worldwide^7–9^. Over the past years, three major angiogenic processes have been well investigated, namely sprouting angiogenesis, splitting (intussusceptive) angiogenesis and vascular mimicry^10–12^. Sprouting angiogenesis is characterized by endothelial cell (EC) activation, enzymatic degradation of basement membrane (BM) and EC migration within the interstitial matrix. Enzymatic degradation of BM increases vascular permeability, which leads to extravasation of proteins and their accumulation in the interstitial matrix forming a new, provisional extracellular matrix (ECM) rich in collagen, fibronectin and laminin^2,13^. Intussusceptive (splitting) angiogenesis is a process in which new blood vessels are formed by the transvascular tissue pillars that develop within capillaries, small arteries and veins, thus expanding the capillary network to grow in itself. This process is rapid and does not involve EC proliferation^11,14–16^. Lastly, vascular mimicry is defined as fluid-conducting, matrix-rich, vasculogenic-like channels formed by highly invasive and genetically dysregulated tumor cells, which transdifferentiate into endothelial-like cells and express endothelial cell phenotype and function^17,18^.

It is well known that the ECM plays a crucial role in the regulation of all the forms of angiogenesis mentioned above, including proliferation of ECs, adhesion, migration, differentiation and lumen formation. Additionally, several studies have reported that survival and proliferation of ECs is strongly dependent on the adhesion of ECs to the ECM. The ECM provides basic structural support and direct signaling functions^13,19–21^, forming a complex three-dimensional network composed of glycosaminoglycans (GAGs), proteoglycans, collagens, fibronectin, laminin and elastin^21,22^. Vascular ECM contains two different compartments: the basement membrane (BM), composed mainly of collagen IV, fibronectin, laminin, heparan sulphate, proteoglycans and nidogens^23^, and the interstitial ECM, comprised primarily of elastic fibers and fibrillar collagen type I (see review by Marchand M.^24^). Although the general importance of ECM for EC migration, proliferation, survival and angiogenesis have been well investigated, the relative importance of specific collagen fibers in supporting these processes is less clear. To the best of our knowledge, little work has been done to investigate the role of ECM collagen fibers in providing guidance cues for EC migration in regenerative angiogenesis.

Over the last decade, the zebrafish model became a powerful model for investigating angiogenesis and tissue regeneration^25^. Due to the optical transparency, high number of offspring, possibility for genetic manipulation, simple maintenance, rapid development and amazing capacity for tissue regeneration, this model has become commonly used in the field^26^. Although the zebrafish seems far removed from humans, they share remarkable similarities at the molecular, physiological and anatomical level^27^. Transgenic zebrafish lines, having fluorescence proteins expressed under specific tissue promotors, enable investigation into the proliferative and migratory behaviors of cells involved in new blood vessel formation *in vivo*^26^. The zebrafish has the potential to completely regenerate many of its tissues, such as the heart, retina, spinal cord, scales and fins^28^. In particular, the caudal fin provides an ideal tissue for investigating vascular regeneration in adult zebrafish due to its simple, thin architecture, relative transparency, accessibility and almost unlimited regenerative capacity^29^. The caudal fin contains segmented bony rays, interray and intraray mesenchymal tissue, filled with blood vessels and nerve axons^28^. It has been shown that the adult caudal fin is regenerated within 30 days and depends on appropriate wound healing, and the creation and progressive redifferentiation of blastemal progenitor cells^29–32^. In the current manuscript, we investigate the contributions of the ECM in the blood vessel formation using the zebrafish fin regeneration assay. We investigated the role ECM collagen fibers play in generating guidance cues for EC migration during regenerative angiogenesis.

## Materials and methods

### Animal care and maintenance

For all animal experiments, adult transgenic zebrafish, aged 10–20 months, were used. Zebrafish were raised and maintained in a water system (Tecniplast ZebTEC facility) at 28.5 °C, a pH of 7.4, conductivity of 500μS and a 14:10h light and dark diurnal rhythm. They were fed bi-daily with live brine shrimp (Special Diets Services, Germany) and dry food (GM-300, Skretting, France). The following transgenic zebrafish lines were used: Tg(fli1a:eGFP) in which ECs are labelled with a green fluorescent protein^33^; and Tg(fli:eGFP)// Tg(colRN:mCherry-NTR) in which ECs express a green fluorescent protein^33^ and cells producing collagen 1α2 fibers (col1α cells) express a red fluorescent protein^34^. All the animal experiments were performed according to the ARRIVE^35^ guidelines, guidelines of the Swiss Animal Welfare Act, and approved by the Veterinary Office of the Canton Bern and Swiss Animal Welfare Ordinance. The study protocol was approved by the Bernese Cantonal Animal Welfare Commission with the corresponding permit issued by Bernese Cantonal Veterinary Office (No. BE67/18). All manipulations were done under anesthesia with Tricaine (MS-222, Sigma-Aldrich), and all efforts were made to minimize suffering. The zebrafish were monitored once daily on swimming behavior, body weight (estimated by eye) and overall appearance. No animals showed signs of pain or distress during the experiment.

### Fin amputation

For the amputation of the caudal fins as well as during subsequent observation periods, zebrafish were anesthetized using 0.04% Tricaine. The caudal fin amputation (approx. 50% lesion size) was performed perpendicular to the cranio-caudal axis of the zebrafish using a razor blade. After amputation, animals recovered and were placed back in the same water system.

### In vivo imaging

Caudal fin regeneration was monitored at several time points up to 30 days post amputation (dpa). At each time point, zebrafish were anaesthetized with 0.04% Tricaine, transferred to the petri dish, immobilized with thin cover glass and recorded by a Leica M205FA stereomicroscope. High-resolution in vivo imaging was performed with the Zeiss LSM880, using a 20 × air objective lens. After monitoring, all fish recovered and were placed back in the same water system. Data were displayed and analyzed using ImageJ software.

### Transmission electron microscopy (TEM)

For transmission electron microscopy (TEM), amputated fins were processed according to standard protocol. Briefly, the fins were fixed in Karnovsky’s solution overnight at 4 °C, washed in sodium cacodylate buffer, dehydrated in a graded series of ethanol, and embedded in EPON resin (Sigma-Aldrich). Blocks were cut at a thickness of 60 nm with an ultra-microtome (Leica), equipped with a diamond knife (Diatome). Sections were mounted on copper specimen grids (Plano) and stained with uranyl acetate and lead citrate for 40 min. The specimens were examined using a Philips TEM CM12 electron microscope.

### Pharmacologic treatments

After the caudal fin amputation, fish were incubated in a water system containing the following compounds: β-Aminopropionitrile (BAPN - 1 mM) or Nifurpirinol (NFP - 4 µM).

#### Β-Aminopropionitrile

β-Aminopropionitrile (BAPN) is an inhibitor of lysyl oxidase and consequently collagen cross-linking leading to reduced collagen production^36^. Prior to the actual experiment, an initial drug optimization experiment was performed in which five different concentrations of BAPN were tested. Based on the literature, fish phenotype and welfare during the above optimization, we decided to use a concentration of 1 mM BAPN with an exposure period of 30 days. Briefly, at day 0, a caudal fin amputation was performed and fish were placed in 1 of 2 tanks - one containing normal fish water (control group) and a second containing 1 mM BAPN (BAPN-treated group). Five fish per group were investigated at nine time points (1dpa, 3dpa, 5dpa, 7dpa, 10dpa, 15dpa, 20dpa, 25dpa, 30dpa). Fish were fed normally and placed in the same tanks during the entire experiment.

#### Nifurpirinol

The zebrafish line, fli:eGFP//colRN:mcherry-NTR, involves the targeted expression of a bacterial nitroreductase (NTR) under a cell‐specific promoter, which reduces an NTR-recognized prodrug into cytotoxic products leading to apoptosis of a targeted cell type. The prodrug used here in conjunction with the NTR system is the nitroaromatic antibiotic, Nifurpirinol (NFP)^37^. In order to select the most suitable drug concentration, a drug optimization experiment was performed in which five different concentrations were tested. Based on the literature, fish phenotype and welfare, we decided to use a concentration of 4 μM NFP for a treatment period up to 30 days. Briefly, at day 0 caudal fin amputation was performed and fish were placed in 2 tanks - one containing normal fish water (control group) and a second containing 4 μM NFP (NFP-treated group). Five fish per group were investigated at nine time points (1dpa, 3dpa, 5dpa, 7dpa, 10dpa, 15dpa, 20dpa, 25dpa, 30dpa) and fed normally. During the entire experiment, the control group was placed in the tank containing normal fish water and the treated group was placed in normal fish water during the day, and in a tank containing 4 μM NFP overnight over the course of the treatment period.

#### Drug treatment: zebrafish embryos

Tg(fli1a:eGFP) and Tg(fli:eGFP)//Tg(colRN:mCherry-NTR) embryos were maintained in E3 medium until 24 h post fertilization (hpf). Control and treated groups were separated accordingly and freshly prepared 1 mM BAPN or 4 μM NFP was added to the designated treatment group starting from 24hpf until 5 days post fertilization (dpf). The control embryos in E3 medium were maintained in the same incubator until 5dpf. The drug solution for the treated groups was replaced every day with freshly prepared drug solution and images from both control and treated groups were obtained.

### Immunofluorescence staining of transversal sections

Fins were harvested at 3dpa and 7dpa and fixed in 4% PFA for 6 h at 4 °C. After fixation, fins were rinsed in H_2_0 and incubated in 15% sucrose overnight at 4 °C. Afterwards fins were embedded in tissue freezing media, (Tissue-Tek O.C.T. Compound; Sakura), cryosectioned at 7 µm thickness using a cryostat CryoStar XN50 (Thermo Fisher Scientific), collected on Superfrost Plus slides (Thermo Fisher Scientific) and allowed to air dry for approx. 1 h at room temperature (RT). Sections were stored in tight boxes at − 80 °C for future use. Before staining, slices were equilibrated to room temperature for 3 h; the area with sections was encircled with Super Par Pen (Daido Sangyo) to keep liquid on the slides and then left for another 10 min at RT to dry. Afterwards slices were permeabilized with 2% BSA/PBS/0.3% Triton for 15 min at RT and blocked with blocking solution (10% goat serum/0.3% Triton/3% milk) for 1 h at RT. Slices were then incubated in a primary antibody diluted in blocking solution overnight at 4 °C. On the next day, fins were washed 3 × 15 min in 0.1% Triton/PBS and incubated with a secondary antibody diluted in blocking solution for 3 h at RT. The fins were washed again and mounted. The following primary antibodies were used: rabbit anti-collagen I (1:250; Santa Cruz Biotechnology; sc-28654), rabbit anti-collagen II (1:200; Sigma-Aldrich; SAB2701915), rabbit anti-collagen IV (1:250; abcam; ab6586), chicken anti-GFP (1:250; aveslabs; GFP-1020) and rat anti-Cherry (1:250; Thermo Fisher Scientific; M11217). The following fluorescently labelled secondary antibodies were used: goat anti-rabbit IgG Alexa Fluor 647 (abcam; ab150079), goat anti-chicken IgY Alexa Fluor 488 (abcam, ab150169) and goat anti-rat IgG Alexa Flour 555 (abcam; ab150158), all diluted 1:500. Sections were imaged with a Zeiss 780 confocal microscope fitted with a 20 × objective 1.0 NA with a dipping lens.

### Quantification of fin regeneration

Structure of the zebrafish caudal fin is considered a 2D-system consisting of the supplying artery and two veins per ray with connecting capillaries^38^. Vascular exchange surface and vessel diameter were analyzed considering that blood vessels are round (cylindrical) structures. Three variables describing the whole zebrafish caudal fin were introduced, namely, “total regenerated area” (TRA), “vascular projection area” (VPA), and “vessel area density” (VAD). TRA designates the area of regenerated caudal fin regardless of its vascularization. VPA is the area of all vessels within the regenerated region as projected on fluorescence images. The commonly used derivative of the two mentioned parameters is VAD where VAD = VPA/TRA. VAD represents the density of the vessel projection area within the regenerated tissue^29^.

### Cell culture of HUVEC

Human umbilical vein endothelial cells (HUVEC) (PromoCell, Heidelberg, Germany) were cultured in Endothelial cell media with Supplement Mix (PromoCell) and cultured at 37 °C, a 5% CO_2_ concentration and saturated humidity.

### Scratch wound migration assay

To monitor EC migration with and without collagen fibers, a scratch wound assay was performed according to the supplier protocol (SARTORIUS). A 96-well plate was first coated with collagen I or collagen IV (Sigma-Aldrich) according to manufacturer instructions, dried and incubated overnight at 2–8 °C. Before plating HUVEC cells, the coated wells were washed with 1xPBS (Phosphate-Buffered Saline, MERCK). Cells were plated at a density of 3.5 × 10^4^ cells per well and grown overnight to confluence. The following day, precise and reproducible wounds were generated using the 96-well IncuCyte Wound Maker (SARTORIUS); cells were washed with 1xPBS and fresh media was added before placing the plate inside the IncuCyte live cell imaging incubator. The software was set to scan the plate every hour up to 3 days. The data was analyzed by three integrated metrics: Wound Width (WW), Wound Confluence (WC) and Relative Wound Density (RWD). These metrics are calculated by custom algorithms that are part of the IncuCyte software package.

### Statistical analysis

Data was analyzed using unpaired t-test in GraphPad Prism v8. The difference was considered statistically significant if p < 0.05 and indicated by asterisks (*p < 0.05; **p < 0.01; ***p < 0.001 and ****p < 0.0001; “n” represents the number of biological replicates.

## Results

### Difference between intact (unamputated) caudal fin and caudal fin during early regeneration

**Figure 1:**
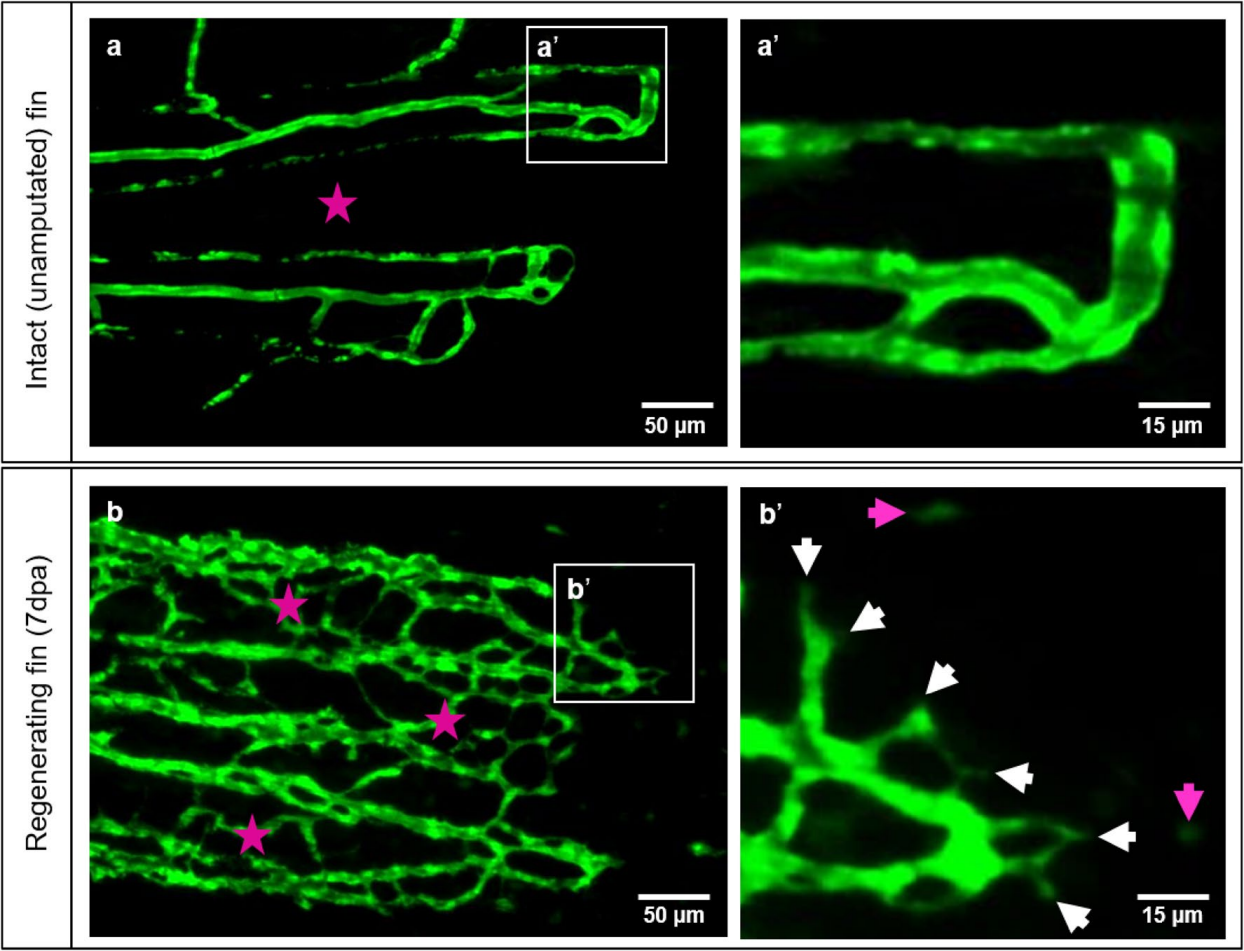
Vascular organization of intact caudal fin vs. early regeneration. Organization of tissue compartments in the intact caudal fin (**a**) versus the regenerating fin (**b**), at 7dpa. a/a’ display smooth and hierarchically well-organized blood vessels (green - reporter transgenic zebrafish line) in which intraray segments do not contain any vessels (asterisk) and capillary sprouts are not detectible (**a’**). (**b/b’**) display dense, well-interconnected capillary meshwork. The intraray region (purple asterisks) contains bridging vessels and at the expanding vascular front multiple sprouts (**b’**, white arrows) and migrating - endothelial cells (purple arrows). Images are acquired by fluorescent reflected light microscope.

**Figure 2:**
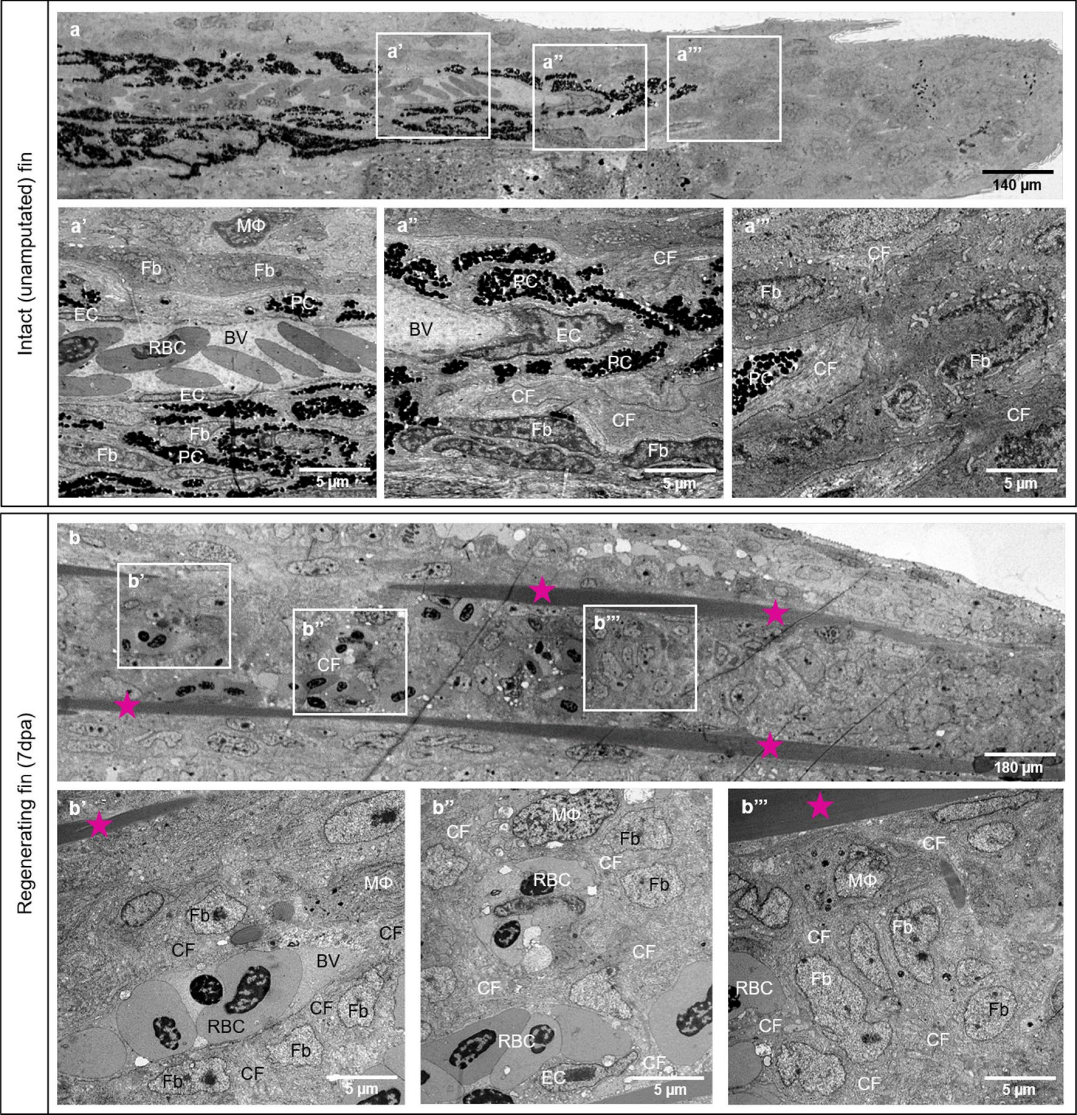
Differences in tissue compartments between the intact (unamputated) caudal fin and fin during early regeneration. Structural differences between intact caudal fin (**a**) and early regeneration at 7dpa (**b**) are demonstrated by transmission electron microscopy. **a-a’’** display perfused and mature blood vessel with quiescent and well-differentiated endothelial cells (ECs), pigment cells (PC) and ECM containing robust collagen fibers (CF), fibroblasts (Fb) and very seldom solitary macrophage (MΦ). In the regenerating fin (7dpa), robust collagen fibers (CF) are arranged into two densely packed stripes tracing the future ray bones (**b**, asterisk). Between the latter, new formed, immature blood vessel (BV) containing red blood cells (RBC) are enclosed by scaffold of CF tracing the direction of blood vessel expansion (**b’**). The CFs are surrounded tightly by Fb and MΦ (**b’**). At the vascular front, next to new formed capillaries containing ECs, clusters or single RBC are detectible (**b’’**). In the blastema, multiple Fb proliferate displaying classical mitotic figures; ECM contains CF and MΦ (**b’’’**). PC are absent during the regenerative phase. Left side - zebrafish trunk, right side - zebrafish tail, images acquired by electron microscope.

Differences in the tissue organization between an intact caudal fin and a caudal fin during early tissue regeneration are displayed (Figs. 1, 2). The intact fin contains simple vasculature with smooth supplying and draining segments, occasionally interconnected; no sprout formation is observed with low capillary density and an avascular intraray region (Fig. 1a, a’). In longitudinal sections, the blood vessels appear mature with quiescent and well-differentiated ECs and are surrounded by robust collagen fiber stripes. The vessels are perfused as indicated by the multiple red blood cells (RBCs) present in the lumen (Fig. 2a, a’). Fin regeneration at 7dpa reveals dense and complex vascular meshwork with well-connected capillaries even within the, normally avascular, intraray spaces (Fig. 1b, asterisk). Multiple capillary sprout formation appears mainly at the expanding vascular front (Fig. 1b’, white arrows). Furthermore, distal to the sprouting front, migrating ECs are visible (Fig. 1b’, purple arrows). In the regenerating tissue (7dpa) different composition of ECM and collagen fibers (CF) were detected (Fig. 2b) with densely packed CF forming a scaffold (Fig. 2b, asterisks). The latter is tracing the regeneration path of new cartilage and bones. Between the scaffolds, the newly formed blood vessels (BV) are embedded: the ECM contains fibroblasts (Fb), macrophages (MΦ), and additional bundles of CF, which line the newly formed capillaries and appear to direct blood vessel expansion. (Fig. 2b’, b’’). The latter are engorged with RBCs and supported by immature ECs (Fig. 2b’’). At the vascular front, polygonal cells with round nuclei, containing heterochromatin and visible nucleolus, reminiscent for activated Fb, as well as MΦ are detectible. (Fig. 2b’’). At the regenerating front, in the blastemal region, multiple proliferating Fb and single MΦ are visible (Fig. 2b’’’).

### Pharmacological and genetic disruption of collagen fiber integrity: effects on regenerative angiogenesis

#### Inhibition of collagen cross-linking by β-Aminopropionitrile (BAPN)

Prior to performing experiments investigating the impact of the collagen cross-linking inhibitor, BAPN, on regenerative angiogenesis, it was first examined whether the effect of BAPN is dependent on the status of wound closure. After the caudal fin amputation, fish were divided into two experimental groups: (i) 2 days in the BAPN solution followed by 5 days in H_2_O or (ii) 2 days in H_2_O followed by 5 days in the BAPN solution. At 7dpa, the vascular phenotype and the dimension of fin regeneration was documented (Supplementary Fig. S1 online). These data demonstrate that the BAPN-induced phenotype is mainly lost when removed after 2dpa (period for wound closure) and the treatment must therefore, be present for the entire experimental period of 30 days.

**Figure 3:**
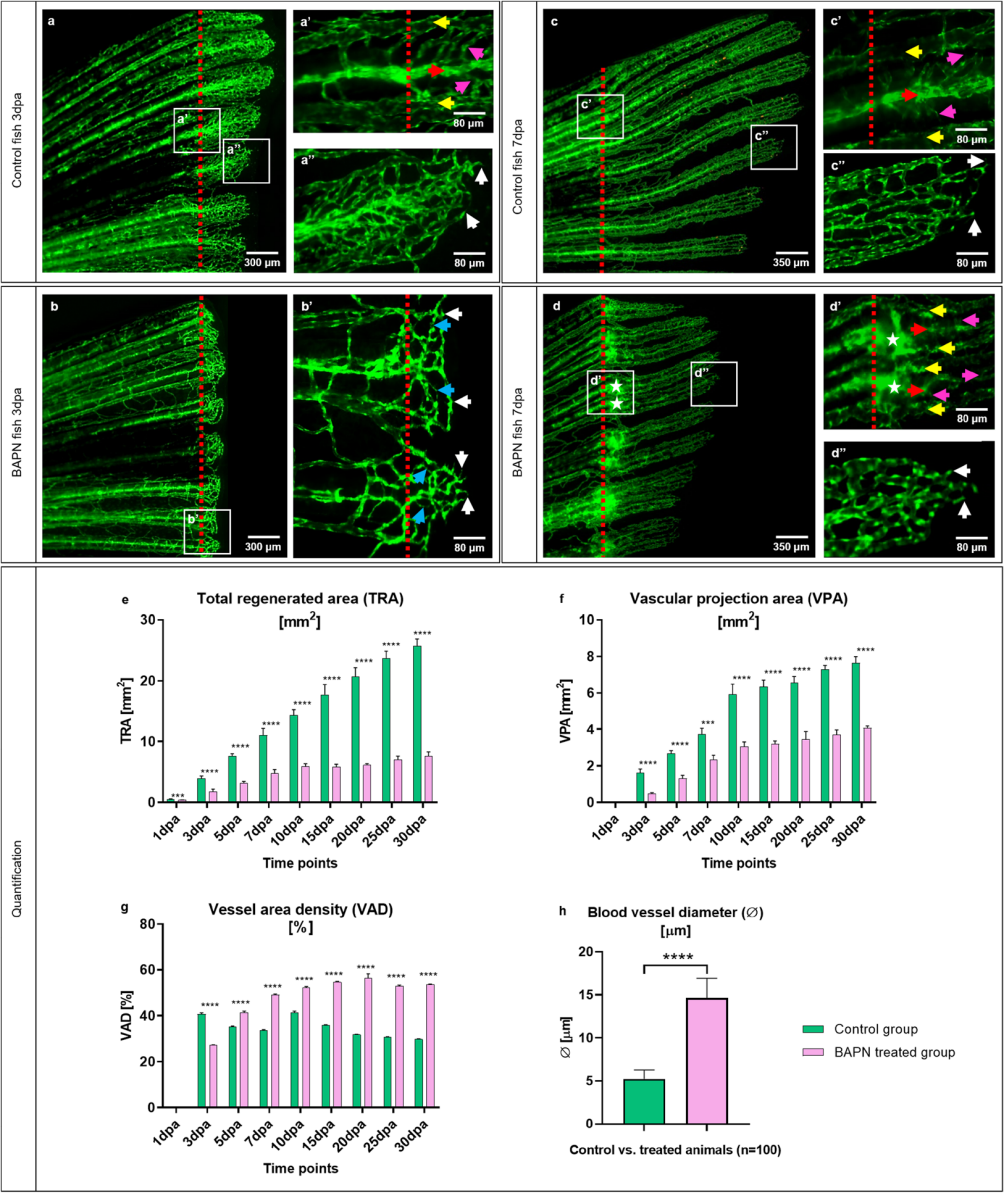
In vivo effects of the collagen cross-linking inhibitor, BAPN, on caudal fin regeneration and neoangiogenesis. Vascular alteration during normal fin regeneration (**a**, **c**) versus BAPN-treated group (**b**, **d**) at 3dpa and 7dpa is documented (green - reporter transgenic zebrafish line). Vessels in the regenerating region of the controls (**a, a’** and **c, c’**, red dotted lines indicate the amputation) appear hierarchically well organized, building a vascular tree with arteries (red arrow), veins (yellow arrows) and connecting capillaries (purple arrows). Capillaries at the vascular front are representing multiple sprouts (**a’’**, **c’’**, white arrows). Shorter regeneration area and respectively modest vascularization in BAPN-treated animals (**b**); hierarchical organization in supplying and draining vessels as well as capillaries is less pronounced. Many capillary connections are very thin and not perfused (**b’**, blue arrows); multiple sprouts are visible at the vascular front (**b’**, white arrows). At 7dpa the regenerating region, respectively the vascular plexus is smaller in comparison to the controls. Balloon-like enlarged blood vessels (detectible as a green sphere) are visible at the site of amputation (**d**, **d’**, asterisks). Those angioma-like structures are connected to the arteries (**d’**, red arrow) and in some cases to the veins (**d’**, yellow arrows). Further distal tiny connecting capillaries are indicated by purple arrows (**d’**): vessel network is disorganized and contains higher number of ECs as indicated by the dense nuclear appearance. Images are acquired with a fluorescent reflected light microscope. Quantification of tissue regeneration and vascularization after inhibition was assessed by three variables: Total regenerated area (TRA = regenerated fin in mm2; **e**), vascular projection area (VPA = vessels within regenerated part in mm2; **f**) and vessel area density (VAD = vessels density within the regenerated part in %; g) during a period of 30 days in control group (green) versus BAPN-treated group (purple). n = 5. **h**) Quantification of lumen dimensions (in μm) between control and BAPN-treated animals. Blood vessel diameter in BAPN-treated animals increases by 70%; n = 100.

**Figure 4:**
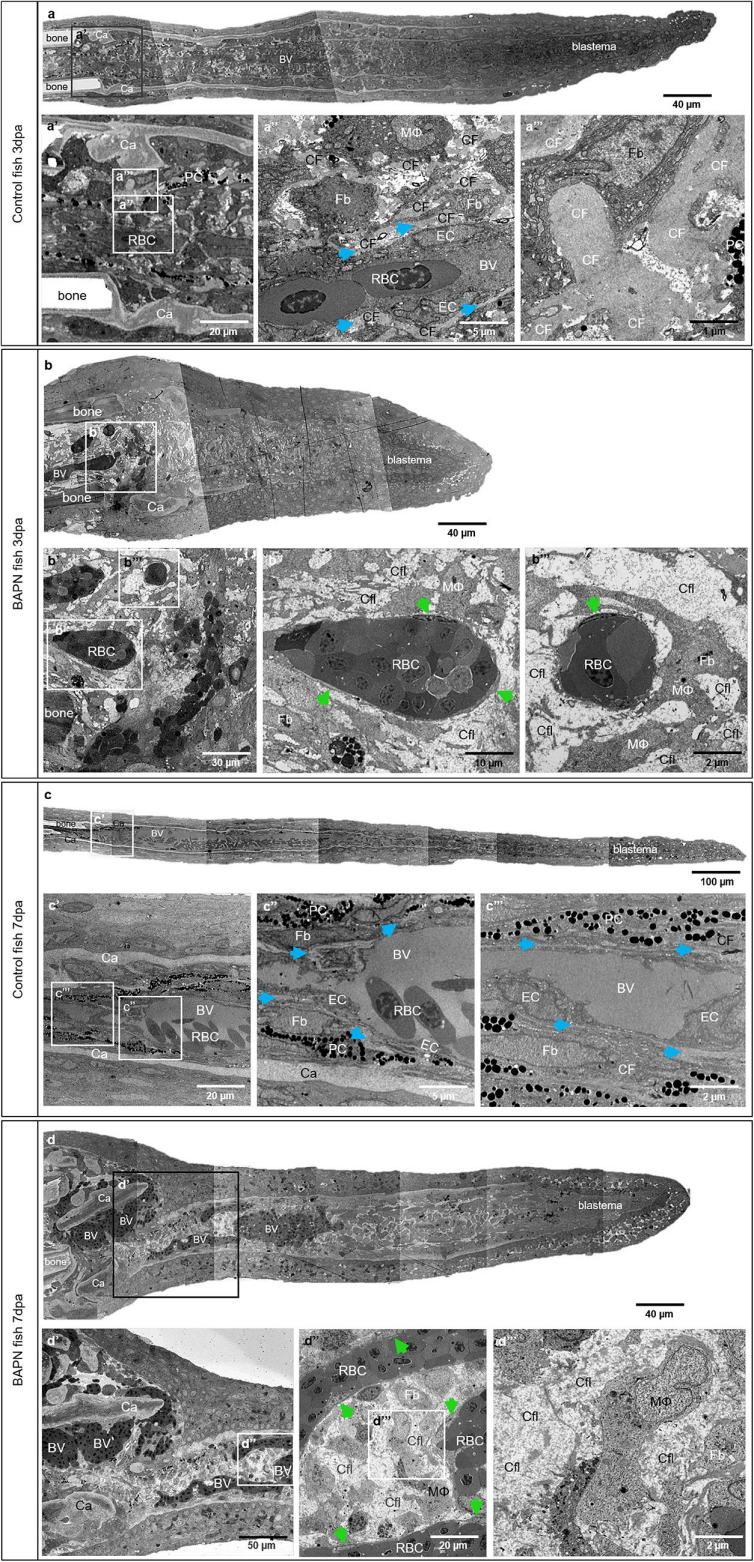
Collagen cross-linking inhibitor BAPN causes structural changes in the collagen content and blood vessel formation. **a**, **b**, **c**, **d** demonstrated reconstructed overview of the fish ray containing bones, cartilage, blood vessels and blastema. Amputation plane by the control group 3dpa (**a’**, **a’’**, **a’’’**) and 7dpa (**c’**, **c’’**, **c’’’**) versus BAPN-treated group at 3dpa (**b’**, **b’’**, **b’’’**) and 7dpa (**d’**, **d’’**, **d’’’**) is shown. **a**–**a’’** display perfused, normal blood vessel with well-differentiated endothelial cells (EC), red blood cells (RBC), solitary pigment cells (PC), bones, cartilage (Ca) and compact, dense ECM containing collagen cross-linked fibers (CF). CF are a natural compartment of the cartilage (Ca) during the early regenerative phase (**a’**); they are also a major component of ECM and located along blood vessel making long sleeves (**a’’**, arrows) or enclosing the fibroblasts (Fb) and tissue macrophages (MΦ). Higher magnification revealed classical collagen cross-linked fibers (**a’’’**). In the BAPN-treated fish, at the 3dpa, the intraray regions are enlarged containing dilated blood vessels with impacted RBCs and extravasal RBCs in front of the latter, i.e. bleeding (**b, b’**). The EC appear seldom (**b’’, b’’’**, arrows), cover only partially the capillaries and the RBCs are in a direct contact with the amorphous ECM (**b’’’**). Classical cross-linked CFs are not detectible; instead, Collagen fibrils (Cfl) appear as amorphous material (**b’’**, **b’’’**). **c/c’** display amputation plane by the control group at 7dpa. Perfused and normal blood vessel is covered by well-differentiated ECs. ECM is compact and dense containing PC, Fb and collagen cross-linked fibers. Collagen is present within the cartilage (Ca) and along blood vessel forming long sleeves (**c’’**, **c’’’**, arrows). **d/d’** display amputation plane by the BAPN-treated group at 7dpa in which balloon-like enlarged blood vessels (BV) containing densely packed RBCs are documented. Those angioma-like structures are perfused and contains partially ECs (**d’’**, green arrows). MΦ and Fb are embedded in ECM with loose and amorphous appearance due to the absent classical cross-linked CF; instead of it Cfl are widely presented (**d’’**, **d’’’**), left side - zebrafish trunk, right side - zebrafish tail, images acquired by electron microscope.

Differences in tissue regeneration and vessel formation (angiogenesis) between the control and BAPN-treated group during early caudal fin regeneration has been documented at 3dpa and 7dpa (Figs. 3, 4). In the control group, the regenerating tissue contains dense capillary meshwork expanding by sprouting at 3dpa (Fig. 3a, a’’). The blood vessels are interconnected, forming a complex network after the amputation plane. Vascular hierarchy is well-defined and structured with supplying arteries, draining veins and connecting capillaries displaying classical in vivo and TEM appearance (Figs. 3a–a’’, 4a–a’’). Morphological observations revealed blood vessels with well differentiated ECs and RBCs at the site of amputation (3dpa), single pigment cells, bones, cartilage and compact, dense ECM-containing collagen cross-linked fibers. CFs were detectible in the cartilage (Fig. 4a, 4a’) and along blood vessels making long sleeves (Fig. 4a’’, arrows). The Fb are surrounded by ECM-containing collagen cross-linked fibers (Fig. 4a’’, a’’’). In the BAPN-treated group (3dpa), the regenerated area and the vascular plexus appear shorter. The blood vessel network is less pronounced and less connected, resulting in reduced vessel density (Fig. 3b, b’). In addition, capillary connections are very thin and likely not perfused (Fig. 3b’, blue arrows) and multiple sprouts are visible at the vascular front (Fig. 3b’, white arrows). The BAPN cross-linking inhibitor dramatically induced morphological changes at 3dpa. The regenerated area is significantly shorter, likely due to impaired blood vessel expansion (Fig. 4b). Intraray regions are enlarged with dilated blood vessels and congested RBCs only partially covered with ECs (Fig. 4b–b’’’). Active Fb with large and abundant cytoplasm were embedded in almost “empty” appearing ECM. In the latter, classical cross-linked CFss are not detectible, instead Collagen fibrils (Cfl), i.e. not cross-linked, appear as amorphous material (Fig. 4b’’, b’’’). Later, at 7dpa (control group), vessels build vascular trees with arteries (Fig. 3c, c’, red arrow), veins (Fig. 3c’, yellow arrows) and connecting capillaries (Fig. 3c’, purple arrows). Vasculature is hierarchically organized and multiple vascular sprouts demarcate the expanding vascular front (Fig. 3c’’, white arrows). Morphologically, the blood vessels are well organized, perfused and covered consistently by well-differentiated ECs. ECM is compact, dense and contains pigment cells, Fb and collagen cross-linked fibers (Fig. 4c–c’’, Supplementary Fig. S2 online). Stripe pattern of collagen deposition could be found in cartilage (Fig. 4c’) and around blood vessels, forming well-defined scaffold sleeves of CF (Fig. 4c’’, c’’’, arrows). The regenerating region and vascular plexus in treated animals at 7dpa are significantly smaller. Balloon-like enlarged blood vessels (detectible as a green sphere) are visible at the site of amputation (Fig. 3d, d’). Those structures are connected to arteries (Fig. 3d’, red arrow) and occasionally to the veins (Fig. 3d’, yellow arrows); between the supplying and draining vessels, multiple connecting capillaries are detected. (Fig. 3d’, purple arrows). Furthermore, the vascular network is hierarchically disorganized and most of the EC nuclei, which exhibit increased density, appear round and not elongated as in the control.

According to the fact, that the zebrafish caudal fin completely regenerates within 30 days, the regeneration process was monitored at nine time points over this period (1dpa, 3dpa, 5dpa, 7dpa, 10dpa, 15dpa, 20dpa, 25dpa, 30dpa). The effect of collagen cross-linking inhibition on fin regeneration was quantified by introducing three variables:

The total regenerated area (TRA = regenerated fin in mm2), vascular projection area (VPA = vessels growth within regenerated fin in mm2) and vessel area density (VAD = vessels density (Fig. 3e–g). TRA in the BAPN-treated group from 1dpa is significantly less relative to control (p < 0.0001). Fin shape and size never returned to pre-amputation levels and the regeneration process slowed after 10dpa with almost no change for the remainder of the experiment (30dpa). In contrast, the control group showed regeneration by linear growth with complete fin regeneration as described previously by Hlushchuk et. al^29^ (Fig. 3e). Vessel growth is not observed at day 1dpa. After the formation of the vascular plexus at 3dpa, a rapid increase in TRA and VPA has been documented in both groups. In the BAPN-treated animals, regeneration and vessel growth are reduced by approximately 70% for TRA (Fig. 3e) and 50% for VPA (Fig. 3f), never reaching the parameters of the controls. Similarly, the VAD is lower at 3dpa (p < 0.0001). After 5dpa, the blood vessel density significantly increased (p < 0.0001) and remained higher versus that of the control group (Fig. 3g). Blood vessel diameter quantification between control and BAPN-treated animals revealed an increase in the lumen dimensions by 70% in the BAPN-treated compared to the control animals (Fig. 3h). Taken together, treatment with the collagen cross-linking inhibitor (BAPN) significantly impairs tissue regeneration by obstructing vessel formation and expansion.

**Figure 5:**
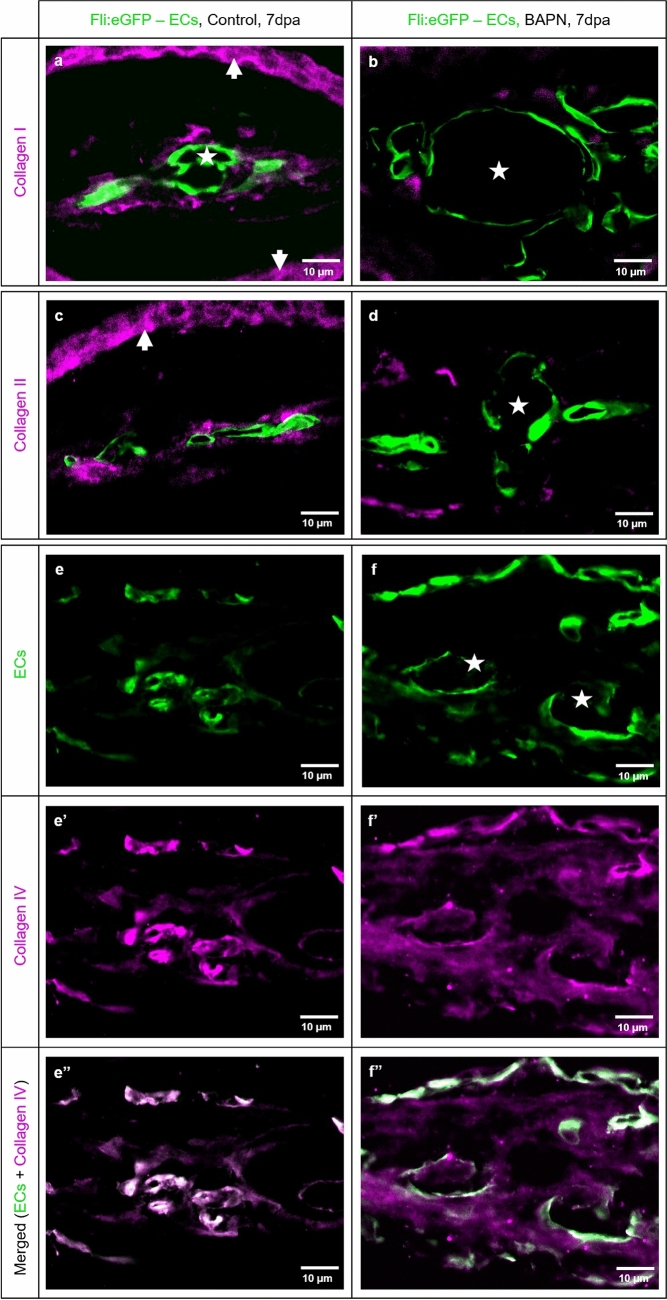
Collagen cross-linking inhibitor BAPN affects collagen I, II and IV accumulation, appearance and distribution. Double immunofluorescence staining revealed the relationship between the new formed blood vessels and collagen I, II, IV distribution in control (left panel: **a**, **c**, **e**, **e’**, **e’’**) vs. BAPN-treated animals (right panel **b**, **d**, **f**, **f’**, **f’’**) at 7dpa. ECs are labeled green and collagen I, II, IV purple. In the control group, the capillaries have classical appearance and collagen I (**a**) and collagen II (**c**) are surrounding the blood vessels as well defined, almost continuous layer. Additionally, collagen I and II stripes are strongly present in the dermal region (arrows). BAPN-treated fish represent dramatically enlarged blood vessels with balloon-like appearance (asterisks). The collagen I (**b**) and collagen II (**d**), when present, appear sparse, as small solitary fragments randomly distributed and not associated with the vasculature and dermal region. In the control group collagen IV demonstrated classical co-localization with the EC as a major component of the basement membrane (**e**, **e’**, **e’’**). In the BAPN-treated animals, collagen IV appears disintegrated and diffuse, without co-localization with the ECs (**f**, **f’**, **f’’**). **e’’** and **f’’** display merged image of ECs and collagen IV corresponding to **e**, **e’** and **f**, **f’** respectively. Images are acquired by confocal microscope.

In addition, the morphological observation at 7dpa revealed balloon-like blood vessels (Fig. 4d, d’) containing densely packed RBCs, partially covered by ECs (Fig. 4d’’, arrows) and activated Fb. Moreover, classical CFs are not detectible and crosslinking is absent (Supplementary Fig. S2 online, asterisk). ECM is represented mainly by Cfl appearing amorphous and surrounding the blood vessel, Fb and MΦ (Fig. 4d’’, d’’’). To visualize and better understand the distribution and structure of CF present at the amputation site 7dpa in the control and BAPN-treated animals, we performed immunofluorescence (IF) staining (Fig. 5). Morphological findings have been confirmed by double immunofluorescence staining performed on the transversal sections at 7dpa. IF demonstrated the relationship between the newly formed blood vessels and spatial distribution of collagen I, II and IV (Fig. 5). Both types of collagen I and II, are wrapping the blood vessels forming a well-defined, almost continuous, layer (Fig. 5a, c). Robust collagen I and II stripes are also characteristic for the dermal region (arrows) in the control animals. The blood vessels appear dramatically enlarged after BAPN treatment with balloon-like appearance (asterisks) and tiny endothelium. Collagen I and collagen II fibers appear sparse with irregular distribution as small solitary fragments (Fig. 5b, d). Dermal expression is minimal. Collagen IV, as a component of the BM, reveals a classical co-localization with the EC in the control group (Fig. 5e–e’’). In the BAPN-treated animals, this was not the case; collagen IV appeared loose, disintegrated and “leaky” without vasculature association (Fig. 5f–f’’).

#### Ablation of collagen 1α2-producing cells by NFP impairs caudal fin regeneration and vascular development

The genetically manipulated zebrafish line fli:eGFP//colRN:mcherry-NTR, incorporates the targeted expression of a bacterial nitroreductase (NTR) under a cell‐specific promoter and treatment with a prodrug (nitroaromatic antibiotic - NFP) that is reduced by NTR into cytotoxic products, leads to apoptosis of the targeted cell type^37^. Before ablating the collagen 1α2 producing cells by NFP, evaluation of NFP delivery into the fish system has been performed analogous to BAPN delivery (Supplementary Fig. S1 online). After the caudal fin amputation, fish were placed for: a) 2 days in the NFP solution followed by 5 days in H_2_O or b) 2 days in H_2_O followed by 5 days in the NFP solution. At 7dpa, fin structure and regeneration capacity were documented (Supplementary Fig. S3 online). Data revealed intake of NFP also after the wound closure (after 2dpa). Based on these results, the exposure time was extended to the entire period of regeneration (30 days). We also treated the zebrafish line (fli:eGFP) that does not encode for NTR with the 4 μM NFP and no effect on the caudal fin regenerative angiogenesis was observed (Supplementary Fig. S4 online). This indicates that NFP induces ablation of collagen 1α2-producing cells specifically through the NTR system.

**Figure 6:**
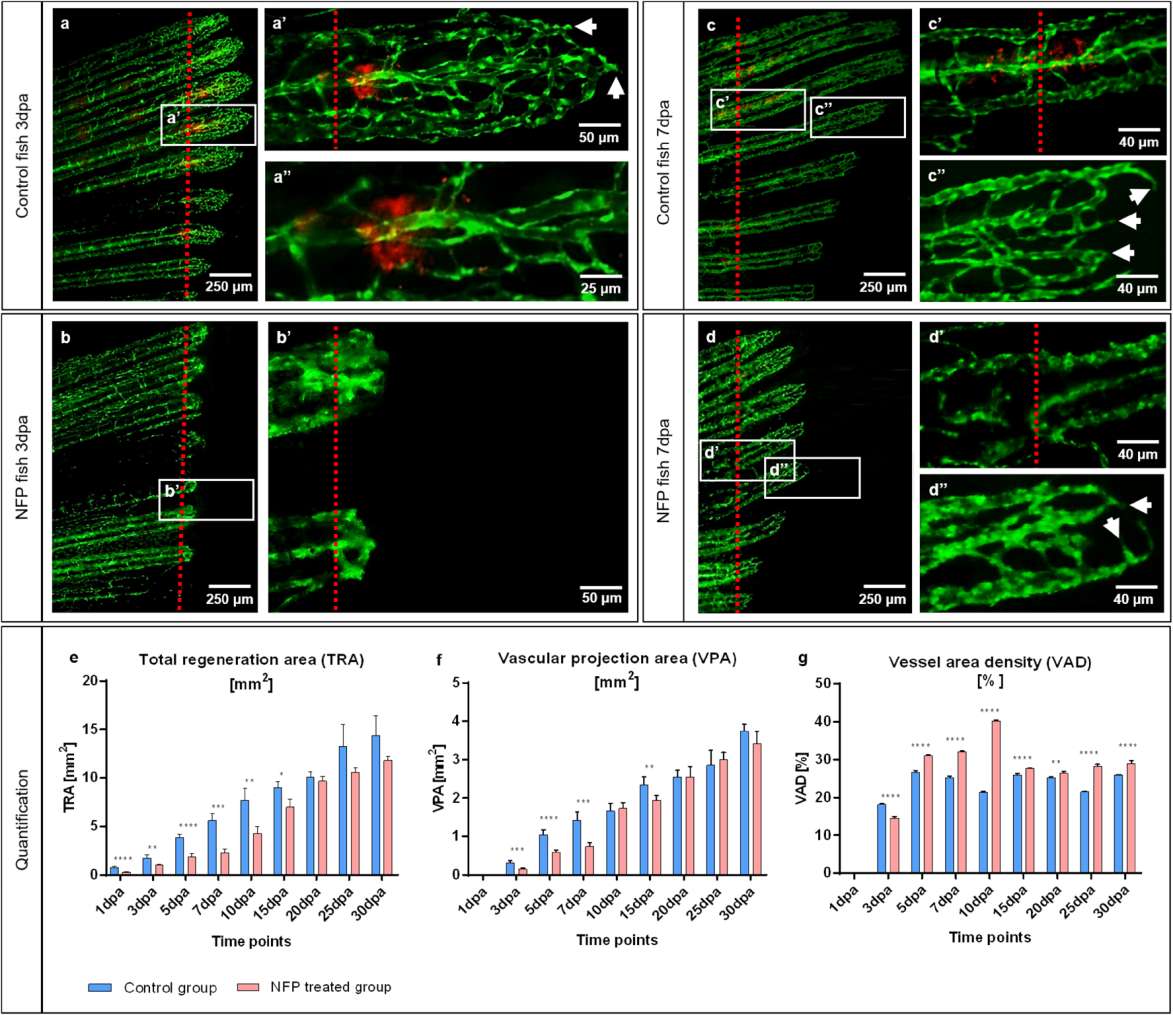
Ablation of collagen 1α2 producing cells by NFP impair caudal fin regeneration and vascular development. In the control animals, at 3dpa and 7dpa, classical tissue regeneration pattern and blood vessel morphology are documented (**a**, **c**). Multiple active cells producing collagen 1α2 (in red) are present adjacent to the amputation line (**a’**, **a’’**, **c’**). Those cells are not detectible in NFP inhibited animals (**b**, **b’**, **d**, **d’**). Regenerative area and vascular plexus appear shorter and underdeveloped in the NFP-treated group at both, 3dpa and 7dpa. Arrows indicated sprouts. Green - ECs, red dotted line - amputation plane, images acquired by fluorescent reflected light microscope. **a’’** part of **a’** at higher magnification. Quantification of the regeneration and vascularization after the elimination of the collagen 1α2 producing cells has been performed by three variables: Total regenerated area (TRA = regenerated fin in mm^2^; (**e**), vascular projection area (VPA = vessels growth within regenerated fin in mm^2^; (**f**) and vessel area density (VAD = vessels density within the regenerated fin in %; g) during the period of 30 days in control group (blue) versus NFP-treated group (red). n = 5.

**Figure 7:**
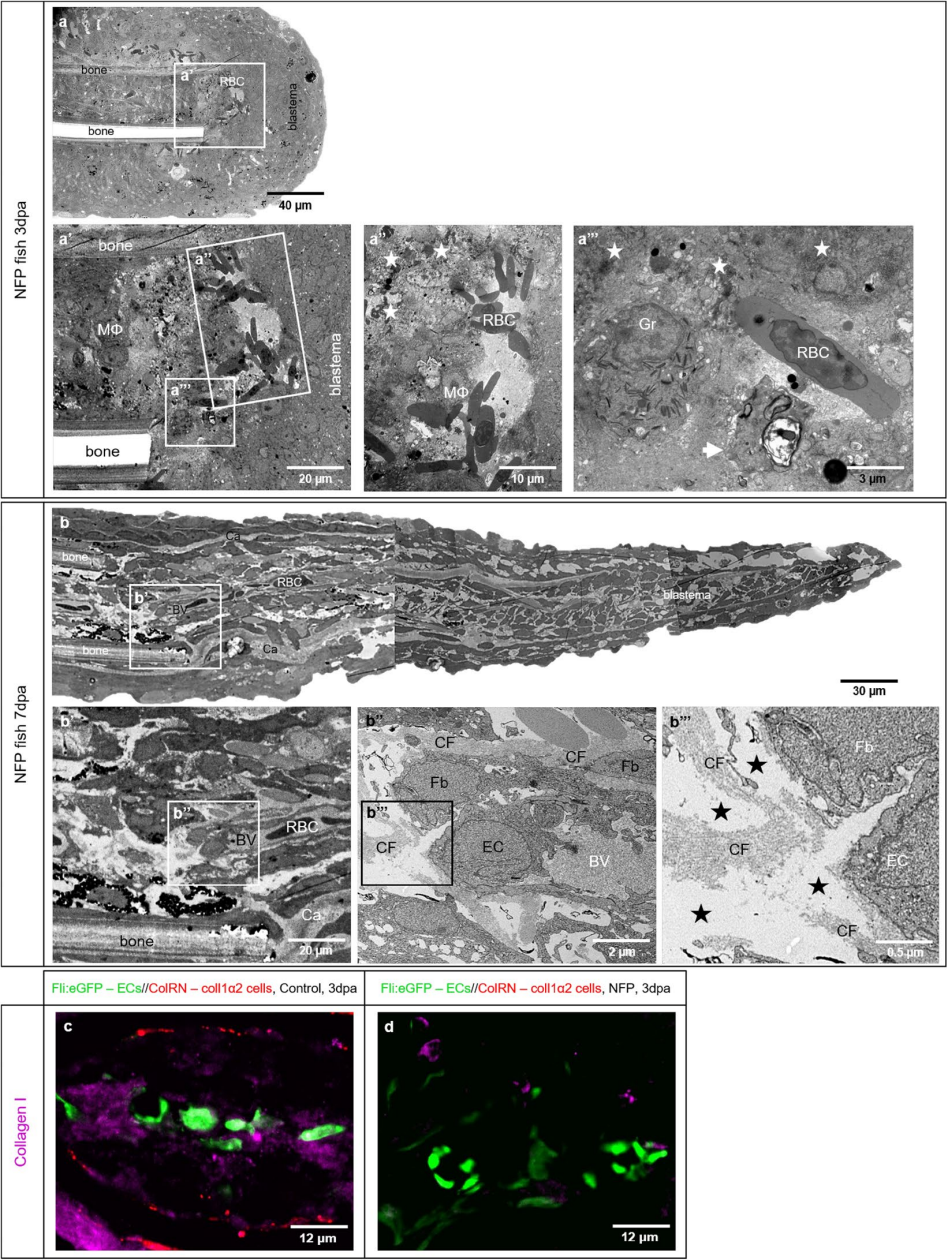
NFP treatment causes collagen deficiency and impairment of caudal fin regeneration. Electron microscopy performed on longitudinal sections revealed severe structural alteration after NFP treatment. 3dpa (**a**) and 7dpa (**b**) represented overview of the fish ray containing bones, RBCs deposition and blastema. **a’**–**a’’’** display clusters and single RBCs that are in direct contact with surrounding macrophages MΦ, and areas of atypical, amorphous ECM. The latter contains disintegrated cells and cell debris (asterisk) as well apoptotic cells (**a’’’**, arrow). In contrast to the controls and BAPN treatment, granulocytes (Gr) and MФ are common features. Classical collagen fibers are not detectible. Amputation plane at 7dpa containing perfused vessels is displayed in **b**; the endothelial coverage is partially interrupted; the EC are activated containing round nuclei and multiple intravascular protrusions. ECM contains less collagen fibers (CF), those do not build classical bundles. The intercellular space is loose, amorphous with irregular distribution representing electron microscopically “empty areas” (**b’’’**, asterisks). Immunofluorescence staining on transversal sections at the amputation site in control animal (**c**) and NFP-treated animal (**d**) at 3dpa are documented. Blood vessels (ECs) are labeled in green, active collagen 1α2 producing cells in red and collagen I purple (**c**). **c** demonstrates intact blood vessels, active collagen 1α2 producing cells, and accumulation of collagen I in the vicinity of the blood vessels. **d** displays blood vessels, very tiny scattered fragments of collagen I and absence of active collagen 1α2 producing cells.

The potential of NFP treatment to inhibit collagen production was documented at 3dpa and 7dpa (Figs. 6, 7). In the control group, normal tissue regeneration and blood vessel expansion, similar to that reported in Fig. 3, was demonstrated (Fig. 6a, c). Red fluorescent cells at the amputation level represent collagen 1α2-producing cells (Fig. 6a–a’’, c, c’). These cells are absent in NFP-inhibited animals (Fig. 6b, d). In addition, the regenerative area and vascular plexus appear shorter and underdeveloped at both, 3dpa and 7dpa. The capillary segments are wider and less inter-connected (Fig. 6b, b’, d–d’’).

Fin regeneration and re-vascularization after the elimination of active collagen 1α2-producing cells was assessed by three variables: The total regenerated area (TRA = regenerated fin in mm^2^) was remarkably smaller by more than 50% during the first 10 days of regeneration (Fig. 6e). Vascular projection area (VPA = vessels growth within regenerated fin in mm^2^) demonstrated the same tendency; and vessel area density (VAD = vessels density within the regenerated fin in %) was higher in the NFP group suggesting larger vessels relative to the controls (Fig. 6g). Growth delay and vascularization were dramatically impaired in the first 10dpa of treatment as demonstrated by the TRA and VPA values. Higher VAD in the NFP-treated group during the entire period of investigation is indicative of substantial dilation of the vascular network in the treated group.

Upon NFP treatment at 3dpa, morphological observations reveled a significantly shorter regeneration area. Classical blood vessel morphology, as well as sprout capillaries, were not observed. Instead, the amputation area contained RBC clusters embedded in atypical ECM containing cell debris and apoptotic bodies, with direct contact to surrounding cells, mainly MΦ and granulocytes (Fig. 7a–a’’’). At 7dpa, there was notable fin tissue regrowth, which is mainly attributable to the large, avascular blastema. The blood vessels are represented in the proximal part and contain immature ECs with rounded nuclei (Fig. 7b’, b’’). ECM appears amorphous and classical CFs are not visible. Triple immunofluorescence staining performed on the transversal sections at 3dpa (Fig. 7c, d) confirmed the absence of active collagen 1α2-producing cells by the NFP-treated animals as well as a dramatic reduction in collagen I production (Fig. 7d).

### Endothelial cell migration is supported by collagen I and IV substrate

**Figure 8:**
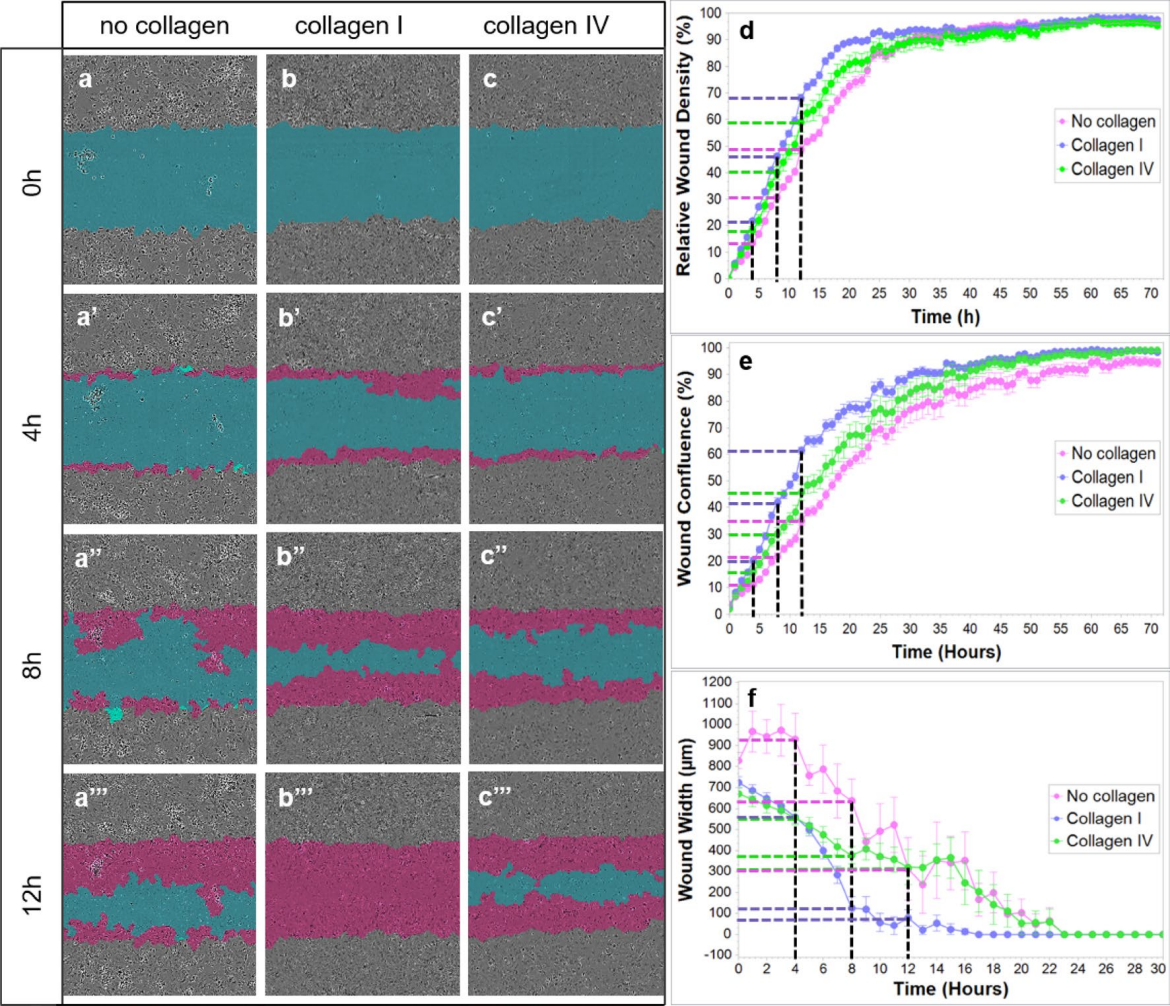
In vitro ECs migration on collagen I and collagen IV substrates. The blue region denotes the scratch wound mask over time (0 h, 4 h, 8 h, and 12 h). ECs migrate into the wound area on non-coated (**a**), collagen I-coated (**b**) and collagen IV-coated wells (**c**). The red area depicts the wound closure by EC expansion over time. The most effective and fast EC migration is observed with the collagen I substrate, followed by collagen IV and lastly, the least efficient, non-coated wells at 0, 4, 8, and 12 h (**a**–**c’’’**; **d**, **e**, and **f**). Relative wound density (**d**), wound confluence (**e**) and wound width (**f**) has been quantified. Cells in the wound area are the most confluent in collagen I-coated wells, followed by collagen IV and non-coated wells (**e**). Blue dashed line - collagen I, green dashed line - collagen IV, purple dashed line - no collagen, black dashed line - time points (4 h, 8 h, 12 h).

EC migration on different collagen substrates was investigated using the in vitro scratch-wound migration assay (Fig. 8). It was apparent that EC migration depends on the matrix used - collagen I- or collagen IV-coated wells vs. and non-coated wells). Our results clearly demonstrate that EC migration is faster and most efficient on collagen I-coated wells with wound closure within 17 h (Supplementary Video S5a online). This is followed by 19 h on collagen IV-coated wells (Supplementary Video S5b online) and 24 h on wells without any matrix support. In addition to slow EC migration, cells were observed to form clusters in the absence of matrix support (Supplementary Video S5c online), which was not observed in cultures supported by either collagen I or collagen IV. The correlation between the type of matrix support and speed of wound closure indicates the importance of ECM collagen composition in EC migration.

Additionally, no differences in EC proliferation status were observed within the wound between wells without collagen and collagen I-coated wells at 4 h and 8 h (Supplementary Fig. S6a–b’ online). Quantification of cell density with the crystal violet assay supports these results as cell number over time did not differ significantly between those plated with (collagen I) or without matrix support at 4 h, 8 h and 12 h (Supplementary Fig. S6c online).

### Pharmacological and genetic disruption of collagen fiber integrity: effects on embryonic zebrafish development

**Figure 9:**
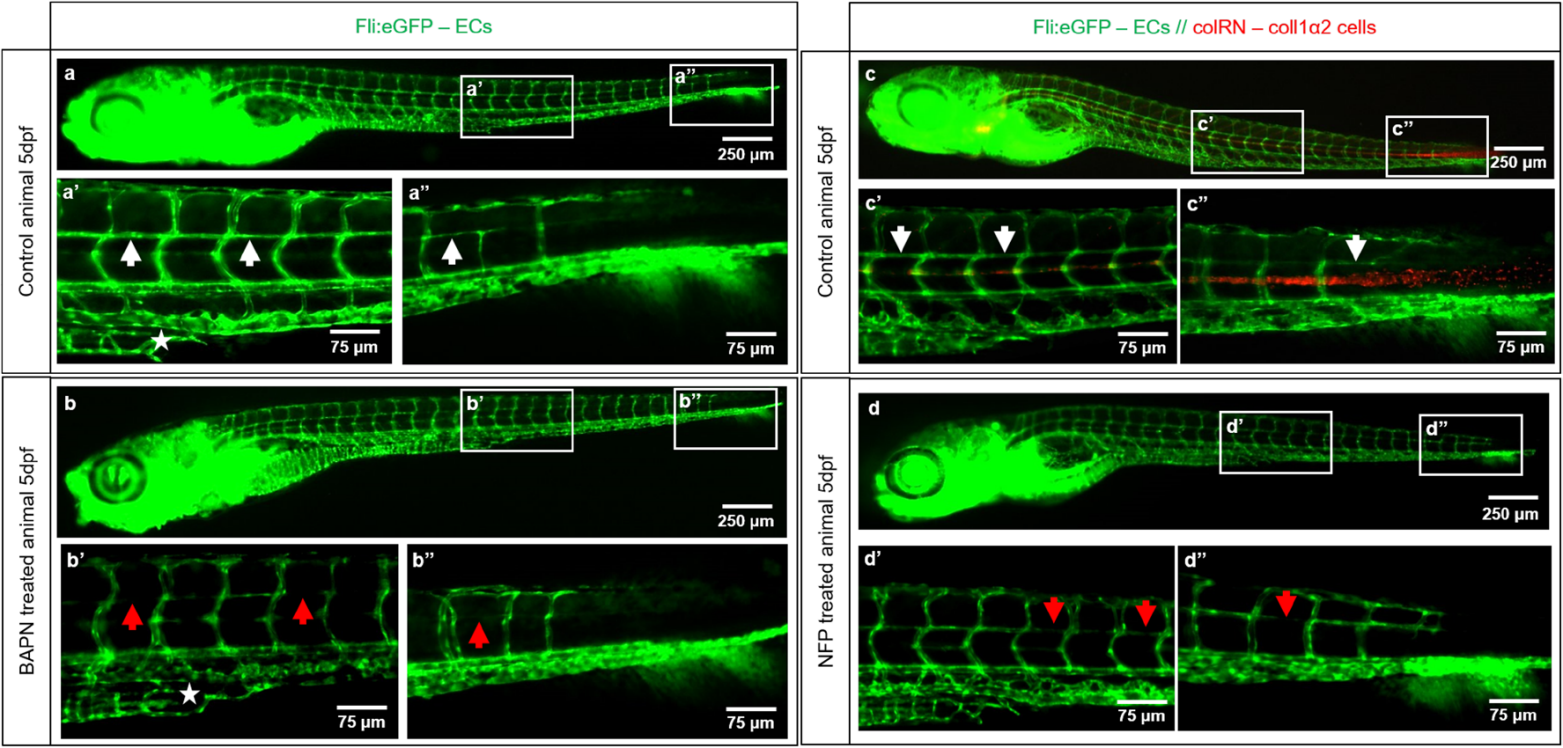
Collagen cross-linking inhibition and reduced collagen 1α2 impair blood vessel formation. Vascular development (ECs - green) in the zebrafish embryo at 5dpf in a control animals (**a**, **a’**, **a’’** and **c**, **c’**, **c’’**) versus BAPN-treated (**b**, **b’**, **b’’**) and NFP-treated (**d**, **d’**, **d’’**) animals is documented. **a’**/**a’’** shows normal caudal plexus formation with horizontal capillaries connecting the intersegmental vessels (ISV; **a’**, **a’’**, white arrow) and subintestinal vein (SIV) (**a’**, asterisk). The BAPN-treated animals show an underdeveloped plexus with absent SIV (**b’**, asterisk) and an impairment in the formation of capillaries connecting ISV (**b’**, **b’’**, red arrows). **c**–**c’’** show normal caudal plexus formation with capillaries connecting ISV (white arrows) and multiple active cells producing collagen 1α2 (in red). Upon NFP treatment, active collagen 1α2 producing cells are absent (**d**–**d’’**) and the formation of horizontal capillaries connecting the ISV is impaired (**d’**, **d’’**, red arrows). ISV - intersegmental vessels, SIV - subintestinal vein; images are acquired by fluorescent reflected light microscopy.

Zebrafish embryos starting from 24 h post fertilization (hpf) were treated for 4 days with i) 1 mM BAPN or ii) 4 μM NFP. BAPN treatment resulted in: (i) retarded caudal vein plexus (CVP) formation with absent subintestinal vein (SIV) at 5 days post fertilization (dpf) (Fig. 9b’, asterisks); (ii) impaired formation, of capillaries horizontally connecting the intersegmental vessels (ISV) in the trunk (Fig. 9b’, red arrows) and in the tail regions (Fig. 9b’’, red arrow). Upon NFP treatment, active collagen 1α2 producing cells are absent and formation of horizontal capillaries connecting the ISV is impaired (Fig. 9d’, d’’, red arrows). In this case, the vascular retardation is not as pronounced compared to that following BAPN treatment, possibly because only one type of collagen (collagen I) is affected.

## Discussion

The ECM plays a major role in angiogenesis. It provides a structural support for blood vessel formation including adhesion to specific ECM components, EC migration, vascular sprouting, and lumen formation^13,39^. Crosstalk between ECM and angiogenesis has been well studied^13,40^, but investigations regarding the role of ECM in guiding EC migration and subsequent vessel formation are lacking. Based on our knowledge, only the ECM component fibronectin has been studied in this capacity^41,42^. Due to the fact that CF are the major components in the ECM^40^ and that collagen IV is one of the major components of the vascular BM^24^, we aimed to investigate the role of CFs in EC migration and vessel formation, specifically, whether these fibers guide EC migration during fin regenerative angiogenesis. To address this question, the well-described zebrafish fin regeneration assay^29^ was employed.

Although caudal fin regeneration in zebrafish has been well described and investigated^43,44^, so far, the tissue organization and compartmentalization of intact (unamputated) caudal fin and caudal fin during early tissue regeneration (7dpa) has not yet been shown. We used a correlative microscopy approach (in vivo light microscopy followed by TEM) to address this question. This study focused on blood vessels formation, organization of the ECM, cell representation and especially on collagen distribution in the regenerating area. During early fin regeneration, vessel formation and the ECM underwent dramatic changes in comparison to the intact fin (Figs. 1, 2). These include changes in (i) vascular complexity containing formation of multiple capillary sprouts and migration of single ECs (Fig. 1); (ii) morphological changes in the ECM and especially of CF composition (Fig. 2). Robust CF bundles are built in the first days after amputation to support cartilage and bone regeneration. They are visible as robust, densely packed stripes tracing the future bone formation (Figs. 2 and 4). More delicate collagen stripes tightly wrap the newly formed blood vessels, building in front of them a scaffold for sprout expansion. MΦ and multiple Fbs surround the scaffold indicating their role in its formation and the proper tissue regeneration. The numerous proliferating Fbs in the blastema also contribute to the expansion of the regenerating fin (Fig. 2).

To elucidate the role of the CFs in the vessel formation, EC migration and tissue regeneration, two pharmacological approaches were used to manipulate collagen fibers: a) inhibition of collagen cross-linking with BAPN and b) selective ablation of collagen 1α2-producing cells using the genetically modified zebrafish line, fli:eGFP//colRN:mcherry-NTR.

Previous studies have reported that BAPN, an irreversible inhibitor of lysyl oxidase (LOX), a key enzyme involved in the cross-linking of collagen in the ECM, results in reduced microvascular density and decreased amounts of cross-linked collagen fibrils in the synovial membrane in a rat model of arthritis^45^. In murine tumour models, BAPN disrupts collagen structure by reducing the amount of collagen cross-linking and inhibiting angiogenesis and micrometastasis^46,47^. To our knowledge, data showing differences in the regenerative potential and vessel formation in the adult zebrafish model, using BAPN, has not been reported.

Consistent with previous studies^45–47^ in vivo models (arthritis rat model, tumor mouse model), our data demonstrated disrupted collagen cross-linking (Figs. 4, Supplementary Fig. S2 online) affecting collagen I, II and IV (Fig. 5) resulting in non-cross-linked Cfl and amorphous ECM (Figs. 4, Supplementary Fig. S2 online). The collagen impairment consequently affected blood vessel formation. The newly formed vessels are much shorter and detectible as a green sphere. The enlarged, balloon-like segments are engorged with RBCs (Figs. 3, 4 and 5). Those spheres are partially perfused and connected mainly to the arteries. It appears that due to malformed CFs, caused by BAPN inhibition, the amputation plane is damaged with a huge accumulation of RBCs without classical blood vessels. EC migration is impaired and disorganized, which results in the establishment of an angioma, i.e. balloon-like blood vessel formation (Figs. 3, 4 and 5). Interestingly, TRA and VPA are decreased, but the VAD increases (p < 0.0001), which means that the fin regeneration is impaired and the relative area covered by the blood vessels within the regenerating area automatically increases (Fig. 3). The data obtained indicate that the collagen cross-linking inhibitor (BAPN) impairs tissue regeneration due to loss of a guidance cues required for EC migration, and dissociation of the basal membrane, both resulting in an abnormal angioma-like vascular phenotype.

Further, we decided to investigate BAPN effect on three collagen types - collagen I, II and IV which have been reported to be essential for angiogenesis and fin regeneration. It is known that collagen I is the major fibrillary collagen type present in ECM with an important role in angiogenesis^13,48^. Collagen II is one of the major components involved in cartilage differentiation and bone formation, and it is strongly expressed in actinotrichia and lepidotrichia during the zebrafish fin regeneration^49^. Collagen IV is one of the major constituents of the BM with an important role in providing stiffness to structural scaffold^24^. Interestingly, we found structural changes in all three collagen types: collagen I and II were deficient with a solitary patchy appearance and not associated with the blood vessels; collagen IV appeared scarce with diffuse cloud-like distribution among blood vessels (Fig. 5). Obtained data indicate that all three investigated collagen types are affected resulting in severe vascular changes and impaired regeneration.

At this point of investigation, it was not possible to specify which collagen was responsible for this vascular guidance. It is known that collagen type I provides structural support and acts as a substrate for cell adhesion and migration^50^ and is the main ECM constituent in which proliferating ECs are exposed to in an injured tissue. It is also important for lumen formation^40^. Therefore, we decided to investigate collagen I using a specific transgenic zebrafish line (fli:eGFP//colRN:mcherry-NTR), by which we are able to reduce the amount of collagen 1α2 during caudal fin regeneration. Firstly, we demonstrated that NFP treatment causes ablation of collagen 1α2-producing cells independently of the fin wound closure (Supplementary Fig. S3 online) and only together with the NTR system (Supplementary Fig. S4 online). Due to the treatment, active cells producing collagen 1α2 fibers are absent (Figs. 6 and 7) resulting in strongly reduced levels of collagen 1α2 fibers (Fig. 7). In vivo observation revealed a deficiency in collagen 1α2-producing cells, shorter regeneration area and an underdeveloped vascular plexus (Figs. 6 and 7). The amputation plane was damaged and accommodates cell debris, apoptotic and disintegrated cells. Intercellular space contained atypical and amorphous ECM with less CFs. Blood vessels are partially covered by ECs and bleeding was observed as cluster of RBCs. Higher numbers of MΦ and Gr were present as expected for clearing tissue debris (Fig. 7). TRA and VPA decreased during the first 10 days of regeneration, meaning the growth delay and vascularization were impaired. We believe that the collagen I scaffold, which traces the direction of blood vessel expansion, is insufficient therefore preventing ECs from expanding properly and impairing angiogenesis and regeneration. In comparison to BAPN treatment, here the effects were not as severe, likely due to the fact that only one type of collagen, among many present in ECM, was targeted.

In both cases, manipulation of CFs results in impaired fin regeneration and angiogenesis, but the dramatic angioma-like phenotype is related to the inhibition of collagen crosslinking. We speculate that the balloon-like structures are formed due to impaired collagen IV and abnormal BM. Collagen I likely serves as a scaffold and specific disruption of collagen I (NFP) results in shortened capillary segments. Collagen IV, as a part of the BM, is important for vascular integrity and normal lumen formation. By over-all collagen cross-linking inhibition, in addition to the shorter segments (collagen I), the disintegration of the collagen IV resulted in insufficient basal membrane and balloon-like vascular phenotype.

It is clear that more than one collagen type is involved in caudal fin regenerative angiogenesis and those different compartments of vascular ECM, basement membrane (collagen IV) and interstitial ECM (collagen I and II) are involved in EC support and migration. This was shown in vitro with collagen I-coated wells promoting the most rapid and efficient EC migration, followed by collagen IV coating and as expected, the slowest migration of ECs occurring on non-coated wells (Fig. 8). Data obtained from the experiments with HUVEC cells used by in vitro scratch wound assay and crystal violet assay indicate that matrix support (collagen I) does not affect the rate of EC proliferation, suggesting that faster closure of the wound can be attributed to cell stretching and migration (Supplementary Fig. S6 online).

We were able to show that CFs have an important role during caudal fin regeneration and vessel formation. Moreover, they can provide guidance for directed EC migration during fin regenerative angiogenesis. The mechanisms behind these interactions, however, still require further investigation. The data obtained from the embryonic zebrafish fin after collagen cross-linking inhibition with BAPN and suppression of collagen 1α2-producing cells by NFP, indicate impaired CVP development and formation of interconnecting vessels between the ISV (Fig. 9). This suggests that collagen support and guidance are important not only during regenerative angiogenesis but also during embryonic blood vessel formation suggesting a widespread role for collagens in any type of angiogenesis. Deeper investigations into the underlying mechanism, however, should be performed in the future. Overall, our study opens new perspectives to understand and control EC migration during physiological and pathological angiogenesis.

## Conclusion

Based on our in vivo and in vitro studies, structural investigations and manipulation of collagen production/cross-linking, we conclude that collagens are key players in regenerative and developmental angiogenesis. CFs (mainly collagen I) provide support and guidance for EC migration and collagen IV is responsible for proper lumen formation and vascular integrity.

## Supplementary Information

Supplementary Information 1.

Supplementary Video 1.

Supplementary Video 2.

Supplementary Video 3.

## Data Availability

All data generated or analyzed in this study are included in this article and the Supplementary Information files.
