# Supplementary material for: Collagen fibers provide guidance cues for capillary regrowth during regenerative angiogenesis in zebrafish
Source: Sci Rep. 2021 Sep 30;11:19520. doi: 10.1038/s41598-021-98852-6 (PMC8484481; doi:10.1038/s41598-021-98852-6)
Supplement: Supplementary file 1 — Supplementary Information 1. [file 41598_2021_98852_MOESM1_ESM.pdf]

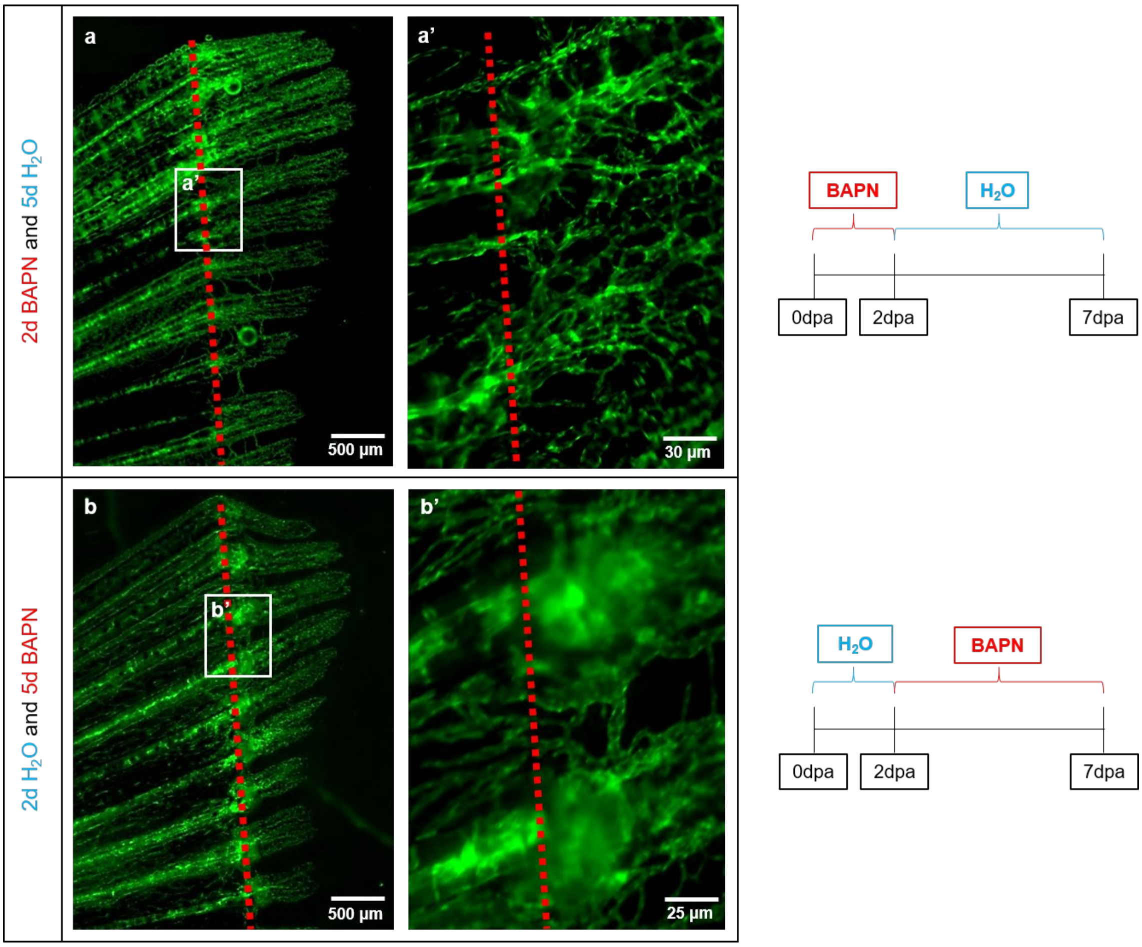

Supplementary Figure S1. Collagen cross-linking inhibitor BAPN is absorbed regardless of wound presence.

To answer the question if BAPN is absorbed and effective only during the phase when the wound is open (2dpa), or is it also active after wound closure, two experiments were performed. After caudal fin amputation (labeled 0dpa), the fish were placed for either (i) 2 days in the BAPN solution followed by 5 days in H_2O (a), or (ii) 2 days in H_2O followed by 5 days in the BAPN solution (b). Both experimental designs demonstrated vascular and fin alterations (see Fig. 3) which were more pronounced in group (ii), likely due to longer exposure to the inhibitor. Detailed descriptions of the effects are depicted in Fig. 3, 4 and 5. Red dotted line – amputation plane; images are acquired by fluorescent reflected light microscope.

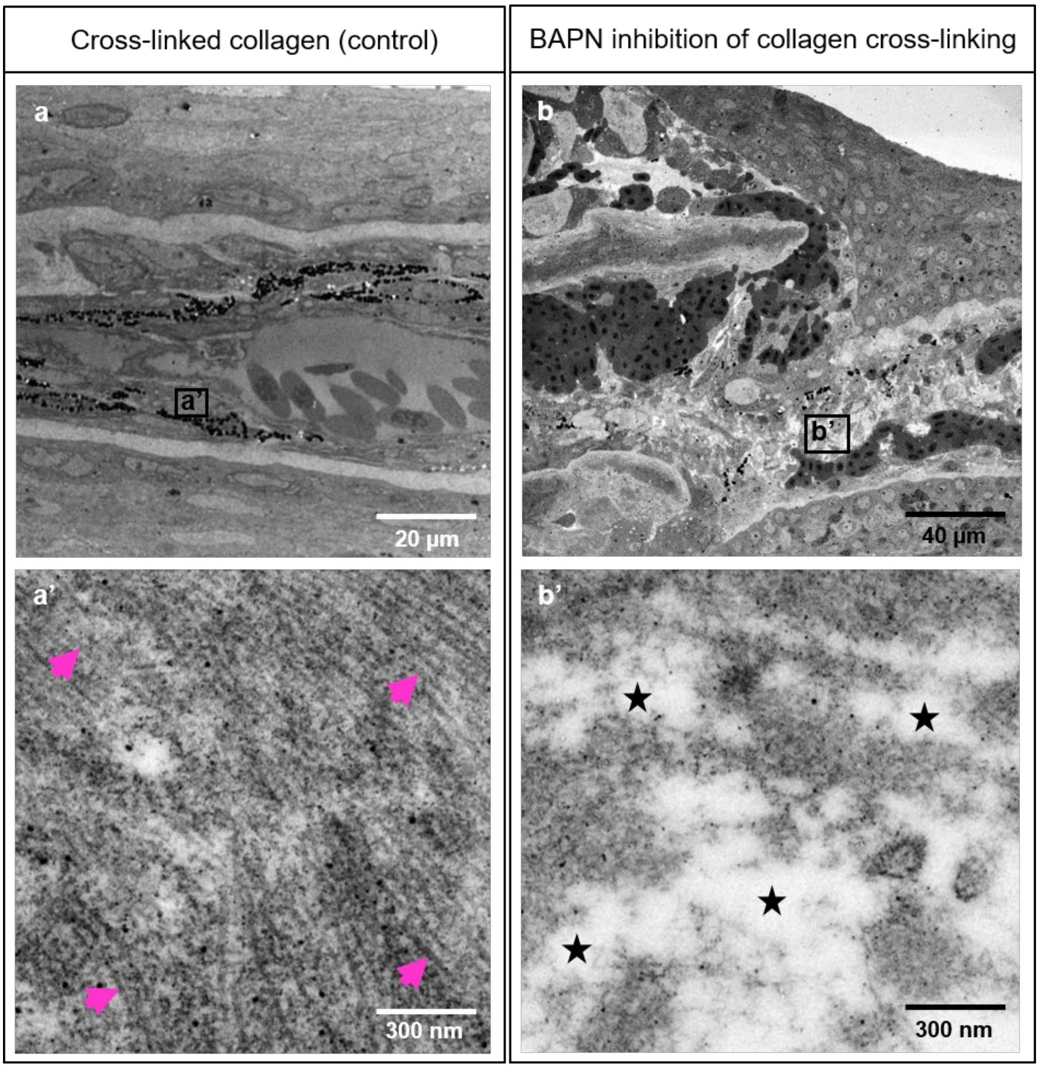

Supplementary Figure S2. Morphological divergence between normal, cross-linked collagen in the untreated fin and collagen in BAPN-treated fin.

Electron microscopy at low magnification demonstrated blood vessels in control (a) and BAPN inhibited animals (b). Higher magnification from the perivascular area revealed classical appearance of cross-linked collagen (a', arrows) versus loose ECM and a reduction in cross-linked fibers with loose collagen fibrils in the BAPN-treated animals (b', asterisk).

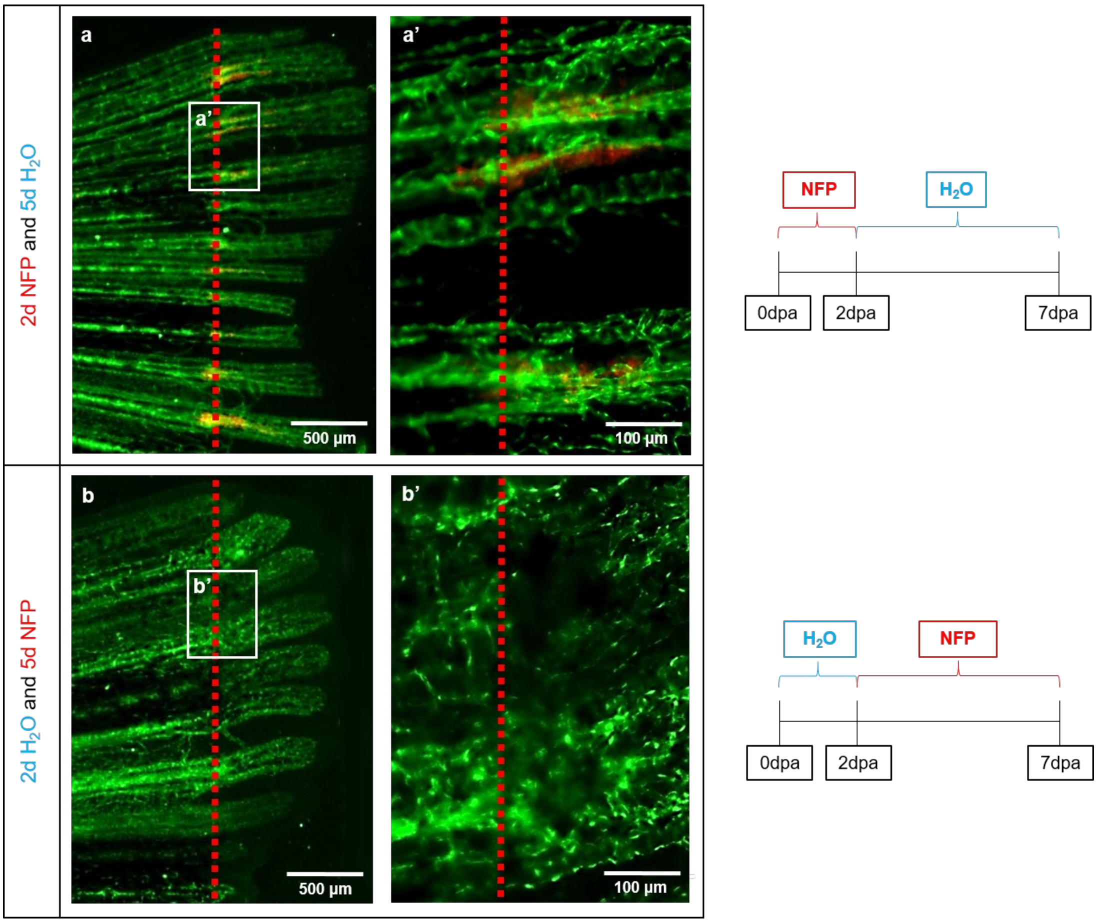

Supplementary Figure S3. NFP induces ablation of collagen 1α2-producing cells independently of wound status.

After the caudal fin amputation (labeled 0dpa), the fish were placed for either: a) 2 days in the NFP solution followed by 5 days in H_2O or b) 2 days in H_2O followed by 5 days in the NFP solution. a/a' display slightly impaired caudal fin regeneration with blood vessels (ECs) in green and collagen 1α2-producing cells in red. Collagen 1α2-producing cells appear to recover after 5 days H_2O without dramatic growth delay and vascular alteration. b/b' show severely impaired caudal fin regeneration with blood vessel malformation and an absence of collagen 1α2-producing cells (red). Red dotted line – amputation plane; images are acquired by fluorescent reflected light microscope.

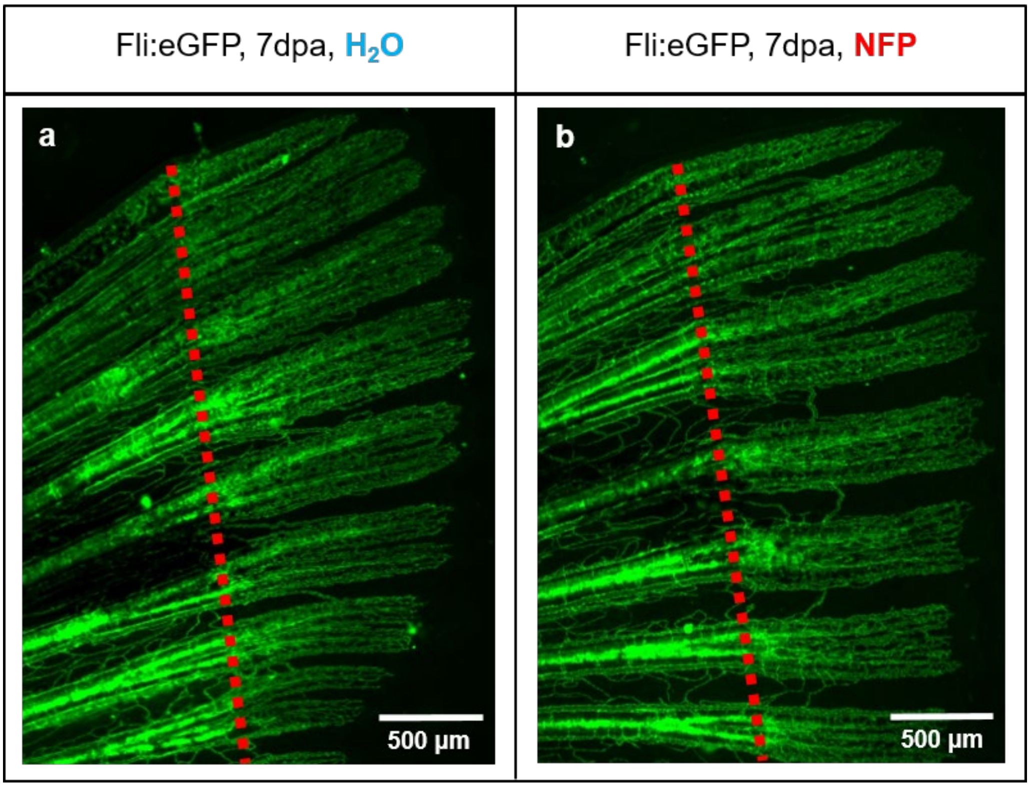

Supplementary Figure S4. Regenerative angiogenesis is not affected by NFP treatment in zebrafish lines that do not carry the NTR system.

Caudal fin regeneration in zebrafish line Fli:eGFP (green ECs) placed into water (a) versus fin regeneration in the presence of NFP (b) at 7dpa. NFP did not affect angiogenesis in lines, which do not carry the NTR system. Red dotted line – amputation plane; images are acquired by fluorescent reflected light microscope.

Supplementary Video S5a-EC migrat

Supplementary Video S5b-EC migra

Supplementary Video S5c-EC migrat

Supplementary Video S5. Scratch would assay to ECs migration on collagen I and IV matrix support.

ECs migrate into the wound on collagen I-coated (a), collagen IV-coated (b) and on non-coated wells (c). The most rapid and effective EC migration is observed on collagen I with wound closure by 17h, followed by collagen IV with wound closure by 19h and lastly, the least efficient migration was observed in non-coated wells with wound closure only by 24h. ECs on non-

coated wells build cluster aggregates (c), which is not detectible by the wells coated with collagen I (a) and collagen IV (b).

To investigate whether collagen I affects endothelial cell proliferation, we conducted additional experiments with HUVEC cells: i) *in vitro* scratch wound assay and ii) crystal violet assay.

i) Scratch wound assay was performed as previously described; after 4h and 8h of incubation following wound induction, cells were fixed and permeabilized in ice-cold 100% methanol for 10 minutes and blocked with blocking solution (1% BSA / PBS / 0.1% Tween 20 for 30 min at RT). Afterwards cells were incubated in the primary antibody rabbit anti-Ki-67 (1:200; Thermo Fisher Scientific; MA5-14520) diluted in blocking solution overnight at 4°C. On the following day, cells were washed 3x5 min in PBS and incubated with the secondary antibody goat anti-rabbit IgG Alexa Fluor 555 (1:500; Thermo Fisher Scientific; A-21428) diluted in blocking solution together with 4',6-Diamidino-2-phenylindole dihydrochloride (DAPI) diluted 1:1000 for 3h at RT. Cells were imaged with a Zeiss LSM880 confocal microscope fitted with a 10× objective 1.0 NA with a dipping lens and analyzed with ImageJ software.

ii) Crystal violet assay- a 96-well plate was first coated with collagen I (Sigma-Aldrich), dried and incubated overnight at 2-8°C. Before plating HUVEC cells, the coated wells were washed with PBS (Phosphate-Buffered Saline, MERCK). Cells were plated at a density of 1×104 cells per well and incubated 4h, 8h and 12h. After each incubation time point, cells were fixed with 0.1% crystal violet (Sigma-Aldrich; V5265) in methanol. After 15 minutes, crystal violet solution was removed, and plates were washed by submerging in tap water until water ran clear. Plates were left to air-dry overnight. Crystal violet was solubilized with 0.1% SDS and absorbance was read at 570nm. Cell number was quantified using the linear equation of a trend line from a crystal violet standard curve generated for HUVEC cell densities ranging from 0-30000 cells per well.

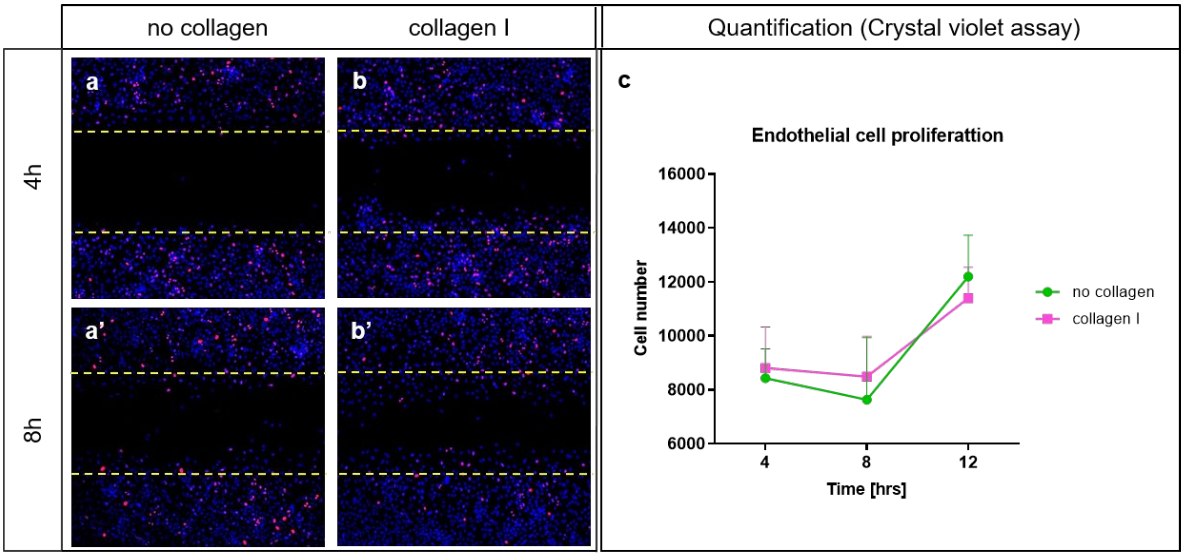

Supplementary Figure S6. ECs proliferation is independent of matrix support.

a-b' depicts EC migration (blue) and Ki67 status (red) after inducing wound (yellow dashed line) on non-coated wells (a, a') and collagen I-coated wells (b, b') at the incubation of 4h (a, b) and 8h (a', b'). ECs migrate and proliferate without difference between the groups. c shows total number of proliferative ECs at 4h, 8h and 12h on non-coated (green) and collagen I (purpura) coated wells. No statistically significant difference upon matrix support; n=8, was documented.
